# Baseline Assessment of Net Calcium Carbonate Accretion Rates on U.S. Pacific Reefs

**DOI:** 10.1371/journal.pone.0142196

**Published:** 2015-12-07

**Authors:** Bernardo Vargas-Ángel, Cristi L. Richards, Peter S. Vroom, Nichole N. Price, Tom Schils, Charles W. Young, Jennifer Smith, Maggie D. Johnson, Russell E. Brainard

**Affiliations:** 1 Joint Institute for Marine and Atmospheric Research, University of Hawaii, Honolulu, Hawaii, 96818, United States of America; 2 2525 Date St. Apt. 3101, Honolulu, Hawaii, 96826–5420, United States of America; 3 Ocean Associates, 1846 Wasp Blvd. Bldg., # 176, Honolulu, Hawaii, 96818, United States of America; 4 Bigelow Laboratory for Ocean Sciences, 60 Bigelow Dr., East Boothbay, Maine, 04544, United States of America; 5 University of Guam Marine Laboratory, Mangilao, Guam, 96913, United States of America; 6 Scripps Institution of Oceanography, University of California San Diego, 9500 Gilman Dr., La Jolla, California, 92093, United States of America; 7 NOAA Pacific Islands Fisheries Science Center, Coral Reef Ecosystem Division, 1846 Wasp Blvd. Bldg. # 176, Honolulu, Hawaii, 96818, United States of America; King Abdullah University of Science and Technology, SAUDI ARABIA

## Abstract

This paper presents a comprehensive quantitative baseline assessment of *in situ* net calcium carbonate accretion rates (g CaCO_3_ cm^-2^ yr^-1^) of early successional recruitment communities on Calcification Accretion Unit (CAU) plates deployed on coral reefs at 78 discrete sites, across 11 islands in the central and south Pacific Oceans. Accretion rates varied substantially within and between islands, reef zones, levels of wave exposure, and island geomorphology. For forereef sites, mean accretion rates were the highest at Rose Atoll, Jarvis, and Swains Islands, and the lowest at Johnston Atoll and Tutuila. A comparison between reef zones showed higher accretion rates on forereefs compared to lagoon sites; mean accretion rates were also higher on windward than leeward sites but only for a subset of islands. High levels of spatial variability in net carbonate accretion rates reported herein draw attention to the heterogeneity of the community assemblages. Percent cover of key early successional taxa on CAU plates did not reflect that of the mature communities present on surrounding benthos, possibly due to the short deployment period (2 years) of the experimental units. Yet, net CaCO_3_ accretion rates were positively correlated with crustose coralline algae (CCA) percent cover on the surrounding benthos and on the CAU plates, which on average represented >70% of the accreted material. For foreeefs and lagoon sites combined CaCO_3_ accretion rates were statistically correlated with total alkalinity and Chlorophyll-a; a GAM analysis indicated that SiOH and *Halimeda* were the best predictor variables of accretion rates on lagoon sites, and total alkalinity and Chlorophyll-a for forereef sites, demonstrating the utility of CAUs as a tool to monitor changes in reef accretion rates as they relate to ocean acidification. This study underscores the pivotal role CCA play as a key benthic component and supporting actively calcifying reefs; high Mg-calcite exoskeletons makes CCA extremely susceptible changes in ocean water pH, emphasizing the far-reaching threat that ocean acidification poses to the ecological function and persistence of coral reefs worldwide.

## Introduction

The uptake of atmospheric carbon dioxide (CO_2_) by seawater and subsequent equilibrium reactions within this ionic medium are part of the complex chemical system often referred to as the marine carbonate system. As atmospheric CO_2_ dissolves in seawater, it forms the weak carbonic acid (H_2_CO_3_), which in turn dissociates into bicarbonate (HCO_3_^−^) and carbonate (CO_3_^2−^) ions, and the associated protons (H^+^). Natural processes including gas exchange, photosynthesis, respiration, calcium carbonate (CaCO_3_) precipitation, and dissolution, influence the distribution of chemical species of the carbonate system as a function of pH [1]. With increased uptake of atmospheric CO_2_ by the ocean, the pH decreases together with CO_3_^2−^ and CaCO_3_ saturation state of seawater, while HCO_3_^−^ increases [2]. However, because the ocean stores roughly 60 times more inorganic carbon than the atmosphere [3], even small changes in the components of the marine carbonate system can have far-reaching implications for surface ocean chemistry, physical properties, individual marine organisms, and marine ecosystems [1, 4, 5].

Since the beginning of the Industrial Revolution, atmospheric global CO_2_ levels have risen by nearly 40% mainly due to the burning of fossil fuels, deforestation, and changes in land usage [6, 7, 8]. It is estimated that elevated CO_2_ concentrations have caused ocean waters to decrease in pH by 0.11 units [9] through the process termed ocean acidification (OA). It is projected that if CO_2_ emissions continue at current rates, atmospheric CO_2_ will reach twice pre-industrial levels by 2065 [10, 11, 12] and ocean surface water pH decrease by 0.14–0.35 units by 2100 [13, 9]. This projected change in ocean water chemistry reduces the pH and the aragonite and calcite (CaCO_3_) saturation states, approaching levels that may not support biogenic calcification but instead drive net dissolution of marine carbonate structures [14, 15, 16, 17]. In addition to calcification, the adverse effects of OA to marine organisms are multiple, affecting other biological and physiological processes, including reproduction, recruitment, development, and growth [18, 19, 20, 21], photosynthesis and respiration [22, 23], acid-base balance and oxygen transport capacity [24,25], behavior, and tolerance to secondary disturbances [26, 27,28].

In shallow tropical marine ecosystems, corals, coralline algae, and other calcifying organisms are responsible for the accretion of biogenic CaCO_3_ that creates the massive, three-dimensional edifices that define coral reef ecosystems and provide the habitat that supports high marine biodiversity. As one of projected consequences of OA to shallow tropical coral reefs, decreased calcification affects carbonate production and consequently net reef accretion rates, potentially impairing ecosystem functionality [29, 30], making coral reefs among the most susceptible marine ecosystems to environmental conditions that impact calcification and/or promote dissolution of CaCO_3_ [31]. Interestingly, the direction and magnitude of the effects appear to be species specific [32, 33].

Calcifying marine macroalgae are a principal component of the carbonate budget on coral reefs, and recent studies suggest they are extremely susceptible to chemical changes associated with OA [16]. Lee and Carpenter [34] estimated that ~50–55% of carbonates present in shallow, tropical marine systems are derived from corals and crustose coralline algae (CCA), while ~35–40% are derived from siphonous green algae (e.g., *Halimeda*, *Udotea*, *Penicillus*, *Rhipocephalus* [35, 36]), and the remaining ~10% are derived from other biogenic calcifiers such as mollusks, echinoderms, and bryozoans [34]. CCA are key components of tropical reef ecosystems [37, 38], often recruiting immediately after disturbances [39] to cement, reinforce, and consolidate carbonate material, often serving as preferred settlement habitat for coral recruits [40, 41], thus, contributing to the buildup, maintenance, and temporal persistence of reef structures [42, 43, 44]. Moreover, species of CCA with skeletal mineralogy composed of high Mg-calcite content are more soluble than organisms with aragonite (corals, *Halimeda*) or calcite (mollusks), and therefore may be the first to be impacted by OA through increased dissolution [45, 46]. In addition, although species-specific, it appears that the extent of damage caused by low pH conditions also depends on the rate of change in the carbonate chemistry [47, 48].

To date, most studies of *in situ* carbonate accretion rates are spatially discrete and conducted on reefs close to urban settlements that are subject to varying levels of anthropogenic impact. Although useful, these data limit our understanding of natural, large-scale spatial patterns, and variability in accretion rates, and fail to provide an accurate baseline that is suitable for modeling or predicting the future effects of OA. To bridge this critical gap, we present the first quantitative baseline of *in situ* net carbonate accretion rates from 78 reefs located on 11 islands in the central Pacific, ranging from high island locales in close proximity to human impacts, to quasi-pristine environs thousands of kilometers away from continental and human influence (see [49]), across various habitats (e.g., lagoons and forereefs), and exposure to wave activity. Using simple and easily-deployed Calcification Accretion Units (CAUs), this study documents and examines: (1) the spatial variation of *in situ* carbonate accretion rates throughout American Samoa and the Pacific Remote Islands Marine National Monument (PRIMNM), (2) the potential association with physical, biological, and oceanographic drivers, and (3) the relational context between observed accretion rates and the composition of the surrounding benthos.

## Materials and Methods

### Study area

**Fig 1:**
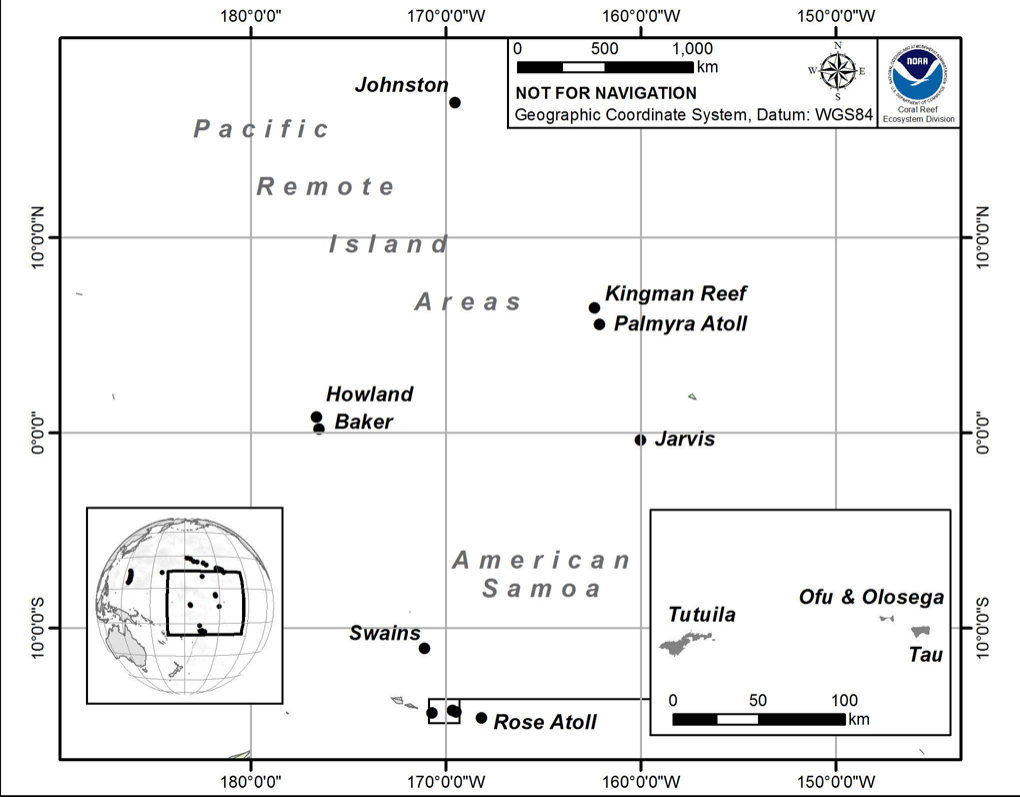
Geographical location of the U.S.-Affiliated Pacific Islands and Atolls where Calcification Accretion Units (CAUs) were deployed and recovered between 2010 and 2012.

Between February and April 2010, the Coral Reef Ecosystem Division (CRED) of the NOAA Pacific Islands Fisheries Science Center (PIFSC) deployed 390 CAUs at 78 reef sites, within two major biogeographical regions (central and south Pacific), including six islands/atolls in the Pacific Remote Islands Marine National Monument (PRIMNM; i.e., Howland, Baker, and Jarvis Islands, Johnston and Palmyra Atolls, and Kingman Reef); and five islands/atolls in American Samoa (i.e., Rose Atoll and Swains, Ta`u, Ofu-Olosega, and Tutuila Islands; Fig 1, Table 1). Study sites spanned ~1700 km E–W and ~3400 km N–S, across a diverse range of geomorphologies, from steep volcanic high islands (e.g., Tutuila, Ta`u, and Ofu-Olosega) to low carbonate islets and atolls (e.g., Howland, Baker, and Jarvis Islands). Oceanographic conditions ranged from intense equatorial and topographic upwelling at Jarvis Island to oligotrophic conditions at many islands (e.g., Rose and Johnston Atolls) [50]; and anthropogenic impact regimes ranged from fishing and chronic coastal runoff (e.g., Tutuila) to lack of any present-day direct human impacts (e.g., Howland and Baker islands, and Kingman Reef) [51].

**Table 1:** Calcification Accretion Unit site locations, depth, reef zone, mean accretion rates, and standard deviation (SD). Availability of benthic cover data from Line-Point-Intercept (LPI) surveys is indicated, Y: yes; N: no.

Archipelago	Island/ Atoll	REA Site	Latitude	Longitude	Depth (m)	Reef zone	Mean accretion rate (g cm^-2^ yr^-1^)	SD	LPI
**Pacific**	**Baker**	BAK-02	0.18839	-176.47994	16.0	Forereef	0.045	0.010	Y
**Remote**		BAK-11	0.19918	-176.48454	10.5	Forereef	0.037	0.006	Y
**Island**		BAK-14	0.20509	-176.47457	16.0	Forereef	0.113	0.014	Y
**Areas**	** **	BAK-16	0.19454	-176.46287	12.0	Forereef	0.092	0.041	Y
	**Howland**	HOW-05	0.80409	-176.62106	11.5	Forereef	0.072	0.017	Y
** **		HOW-11	0.79882	-176.62025	13.5	Forereef	0.070	0.015	Y
** **		HOW-12	0.80924	-176.61068	12.3	Forereef	0.069	0.021	N
** **		HOW-13	0.81962	-176.61619	12.2	Forereef	0.131	0.027	N
** **	** **	HOW-14	0.81463	-176.62386	14.0	Forereef	0.068	0.008	Y
** **	**Jarvis**	JAR-01	-0.36787	-159.97919	15.5	Forereef	0.201	0.033	Y
** **		JAR-07	-0.37611	-160.01393	13.0	Forereef	0.061	0.019	Y
** **		JAR-08	-0.36314	-159.99139	13.5	Forereef	0.106	0.020	Y
** **		JAR-10	-0.38128	-159.97264	13.5	Forereef	0.077	0.050	Y
** **	** **	JAR-11	-0.36902	-160.00819	13.0	Forereef	0.075	0.023	Y
** **	**Johnston**	JOH-09	16.72862	-169.48573	7.9	Lagoon	0.011	0.003	Y
** **		JOH-10	16.76337	-169.51201	14.4	Lagoon	0.006	0.002	Y
** **		JOH-11	16.72154	-169.52430	11.4	Lagoon	0.043	0.016	Y
** **	** **	JOH-12	16.74766	-169.52396	11.0	Lagoon	0.016	0.004	Y
** **	**Kingman**	KIN-03	6.39029	-162.36066	11.0	Lagoon	0.064	0.006	Y
** **		KIN-04	6.43872	-162.38824	15.0	Forereef	0.115	0.019	Y
** **		KIN-05	6.39325	-162.34746	13.0	Lagoon	0.058	0.028	Y
** **		KIN-07	6.40219	-162.38522	10.0	Lagoon	0.112	0.025	Y
** **		KIN-10	6.42041	-162.37955	12.8	Lagoon	0.085	0.019	Y
** **		KIN-11	6.38196	-162.34638	13.5	Forereef	0.106	0.013	Y
** **		KIN-13	6.38220	-162.38406	12.0	Forereef	0.084	0.019	Y
** **	** **	KIN-16	6.39240	-162.34210	7.0	Lagoon	0.055	0.030	Y
** **	**Palmyra**	PAL-01	5.86802	-162.06927	14.0	Forereef	0.048	0.021	Y
** **		PAL-05	5.89582	-162.13795	15.0	Forereef	0.110	0.029	Y
** **		PAL-11	5.88343	-162.13340	15.0	Forereef	0.061	0.008	Y
** **		PAL-12	5.89713	-162.10785	14.5	Forereef	0.059	0.013	Y
** **		PAL-19	5.86630	-162.10956	14.5	Forereef	0.109	0.019	Y
** **		PAL-21	5.89556	-162.08600	13.5	Forereef	0.039	0.009	Y
** **		PAL-25	5.86384	-162.03055	15.0	Forereef	0.078	0.004	Y
** **	** **	PAL-26	5.86414	-162.12698	15.0	Forereef	0.086	0.019	Y
**American**	**Ofu-**	OFU-01	-14.16445	-169.65573	14.0	Forereef	0.115	0.023	Y
**Samoa**	**Olosega**	OFU-02	-14.18511	-169.67573	13.5	Forereef	0.101	0.029	Y
** **		OFU-03	-14.18649	-169.66021	14.5	Forereef	0.102	0.027	Y
** **		OFU-04	-14.17766	-169.64950	12.0	Forereef	0.098	0.017	Y
** **		OFU-06	-14.17419	-169.68197	13.5	Forereef	0.087	0.009	Y
** **		OFU-09	-14.15764	-169.67424	10.5	Forereef	0.079	0.015	Y
** **		OLO-01	-14.16854	-169.60783	14.5	Forereef	0.113	0.032	Y
** **		OLO-04	-14.18173	-169.62661	12.5	Forereef	0.099	0.024	Y
** **	** **	OLO-05	-14.16343	-169.62465	11.0	Forereef	0.069	0.006	Y
	**Rose**	ROS-01	-14.53946	-168.14550	12.5	Forereef	0.152	0.019	Y
		ROS-03	-14.55480	-168.14655	13.5	Forereef	0.175	0.025	Y
** **		ROS-04	-14.55966	-168.15999	12.5	Forereef	0.189	0.023	Y
** **		ROS-06	-14.53641	-168.16548	14.5	Forereef	0.095	0.030	Y
** **		ROS-08	-14.53789	-168.15330	9.8	Lagoon	0.028	0.017	Y
** **		ROS-09	-14.55125	-168.16031	5.5	Lagoon	0.013	0.004	Y
** **		ROS-19	-14.54910	-168.13785	14.0	Forereef	0.181	0.042	Y
** **		ROS-23	-14.54216	-168.17235	13.5	Forereef	0.132	0.023	Y
** **	** **	ROS-25	-14.52932	-168.15348	10.0	Forereef	0.132	0.034	Y
** **	**Swains**	SWA-01	-11.06832	-171.08118	15.0	Forereef	0.139	0.029	Y
** **		SWA-03	-11.05769	-171.09142	14.5	Forereef	0.089	0.020	Y
** **		SWA-07	-11.05098	-171.06581	15.5	Forereef	0.104	0.022	Y
** **		SWA-08	-11.04569	-171.07708	16.0	Forereef	0.076	0.035	Y
** **	** **	SWA-16	-11.05074	-171.09223	12.5	Forereef	0.093	0.023	Y
** **	**Tau**	TAU-02	-14.25171	-169.44617	12.0	Forereef	0.082	0.012	Y
** **		TAU-04	-14.21240	-169.44066	12.5	Forereef	0.097	0.017	Y
** **		TAU-07	-14.22730	-169.41833	13.0	Forereef	0.094	0.010	Y
** **		TAU-08	-14.26240	-169.47480	13.5	Forereef	0.110	0.012	Y
** **		TAU-09	-14.24573	-169.50659	12.8	Forereef	0.100	0.021	Y
** **		TAU-11	-14.21723	-169.51281	14.5	Forereef	0.064	0.010	Y
** **	** **	TAU-12	-14.25756	-169.50101	12.0	Forereef	0.072	0.008	Y
** **	**Tutuila**	TUT-01	-14.28354	-170.63782	13.0	Forereef	0.070	0.019	Y
** **		TUT-02	-14.27780	-170.60723	13.0	Forereef	0.048	0.006	Y
** **		TUT-05	-14.25169	-170.62309	15.0	Forereef	0.043	0.005	Y
** **		TUT-06	-14.32810	-170.83183	14.0	Forereef	0.056	0.011	Y
** **		TUT-08	-14.29167	-170.78042	15.0	Forereef	0.043	0.012	Y
** **		TUT-09	-14.33608	-170.70438	9.0	Forereef	0.069	0.020	Y
** **		TUT-10	-14.31101	-170.69303	14.0	Forereef	0.073	0.024	Y
** **		TUT-13	-14.26055	-170.71205	15.0	Forereef	0.053	0.008	Y
** **		TUT-14	-14.25334	-170.65219	14.5	Forereef	0.053	0.009	Y
** **		TUT-16	-14.28532	-170.56407	14.0	Forereef	0.058	0.014	Y
** **		TUT-17	-14.24600	-170.57196	13.5	Forereef	0.088	0.026	Y
** **		TUT-19	-14.28319	-170.72825	15.5	Forereef	0.050	0.011	Y
** **	** **	TUT-22	-14.36588	-170.76284	14.0	Forereef	0.078	0.017	Y

### Carbonate accretion and community structure

**Fig 2:**
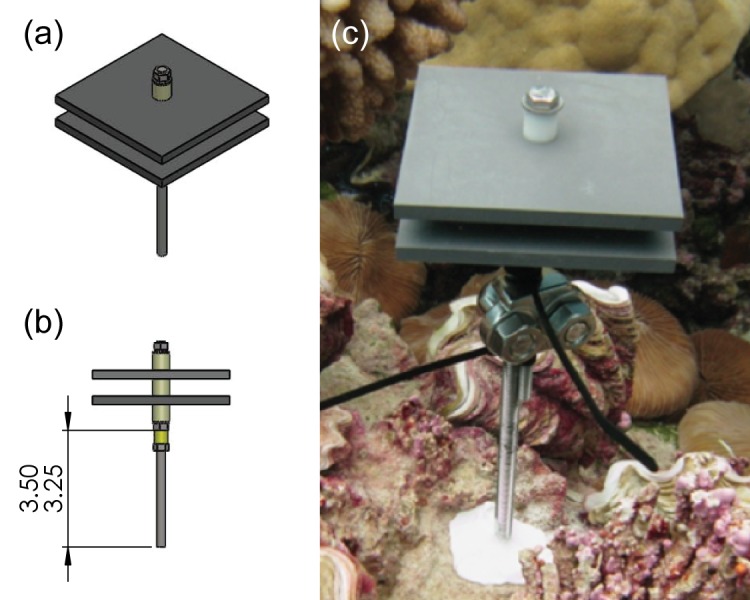
CAU assembly unit: a) oblique view, b) side view, and c) in-situ image of deployed CAU unit. (Photo and figure credit: Coral Reef Ecosystem Division, NOAA).

Each CAU assembly comprised two 10 cm × 10cm (100-cm^2^) polyvinyl chloride (PVC) plates separated by a 1 cm plastic spacer and mounted on a stainless steel all-thread rod (Fig 2). Each PVC plate was sanded to provide a non-glossy surface suitable for permanent attachment and settlement of marine propagules. These assemblies were attached to stainless steel stakes installed into hard carbonate or basalt reef substrate at depths of 5.5–15 m at permanent CRED benthic, Rapid Ecological Assessment (REA) survey sites. Five CAUs were installed at each site, with each CAU being positioned approximately 10 cm above the substrate with a spacing of 0.5–3 m between each CAU. CAUs were typically installed at a minimum of 5 sites per island (2 islands/atolls had only four) and sites were spread out across the forereef and lagoon sites (where possible) for representative spatial coverage.

CAUs were deployed for a ~2-year period and were recovered during the February–May 2012 CRED-led Pacific Reef Assessment and Monitoring Program (Pacific RAMP) research cruise. During recovery, each CAU was placed in a Ziploc^®^ bag to minimize the loss of attached organisms or calcified material during transport to the shipboard laboratory onboard the NOAA ship *Hi`alakai*. In this laboratory, CAUs were rinsed in salt water to remove mobile fauna and sediment/sand, and then frozen at –5°C for preservation during transportation to the laboratory in Honolulu (7–60 days). In the Honolulu laboratory, each CAU was disassembled and each plate submerged in a shallow (5 cm) basin of salt water; the upper and lower surfaces of both plates were photographed to characterize and quantify the settled early successional benthic community.

Subsequently, plates were dried at 60^°^ C for 2–5 days, repeatedly weighed throughout the drying process, and classified as dry when the difference in weight between sequential weighings was less than 0.1g. After drying, each individual plate was submerged in 5% HCl for 24 hrs or until all CaCO_3_ had dissolved. During the dissolution process, plates were periodically agitated (every 1–8 hrs) to reduce the boundary layer dissolution impediments, and large pieces of CaCO_3_ were crushed using a pestle to speed dissolution. As the HCl solution was neutralized by the CaCO_3_ dissolution (indicated by the absence of gas bubbles), additional HCl was added to complete the dissolution process. Often, the addition of acid was repeated several times in a 24–72 hr period until all CaCO_3_ was removed. The remaining fleshy tissue was scraped onto pre-weighed 11 μm cellulose filter paper, vacuum filtered along with all 5% HCl supernatant from the dissolution process, dried at 60^°^C (until constant weight using the same dryness criteria above; 48 hours minimum), and weighed. Finally, the clean, scraped, and dried CAU plates were re-weighed, and the mass of CaCO_3_ was determined by subtracting the combined weight of the fleshy tissue and PVC plates from the initial dry weight of the CAU prior to dissolution. To determine the rate of CaCO_3_ accretion, the mass of CaCO_3_ was normalized for surface area of each CAU (400 cm^2^—accounting for all upper and lower plate surfaces) and the amount of time in days that each CAU was deployed, rendering a measure of net CaCO_3_ accretion in units of g cm^-2^ yr^-1^.

Community composition and percent cover of all taxa recruiting to and settling on the CAUs were characterized based on image analysis of each of the 4 CAU plate surfaces, implementing the software PhotoGrid 1.0 (25 stratified random points analyzed per surface). Sessile organisms were classified into ecological functional groups as follows: calcified macroinvertebrates, corals, crustose coralline algae (CCA) (i.e., Family Corallinaceae), encrusting macroalgae, *Halimeda* spp., calcified macroalgae, other calcified algal crusts (i.e., Family Peyssonneliaceae), algal turf assemblages, fleshy macroalgae, and macroinvertebrates (Table 2). For most of the taxa recruiting to and settling on the CAUs, the polymorph of CaCO_3_ is known [52, 53, 54] (Table 2). Thus, based on image analysis of each CAU plate, the relative percent cover contribution for each CaCO_3_ polymorph (aragonite, calcite, or high Mg-calcite) on the CAU plates was calculated by categorizing the calcifying taxa according to their mineralogy, following Price et al. 2010 [29].

**Table 2:** Functional group classification and mineralogical exoskeletal composition of the taxa comprising the benthic communities at study sites and recruited to the CAU plates. NC: non-calcifying.

Functional group	Taxa	CaCO_3_ skeleton mineralogy
Calcified invertebrate	Calcified tubeworms	Calcite
	Barnacle	Calcite
	Entoproct	Calcite
	Encrust/branched bryozoan	Calcite
	Vermetid, bivalve	Calcite/Aragonite
	Other calcified Invert	Calcite
CORAL	Scleractinian coral	Aragonite
	Hydrocoral	Aragonite
CCA	Encrusting coralline algae	High Mg-Calcite
	Branching coralline algae	High Mg-Calcite
Calcified algal crusts	*Palmophyllum*	Calcite
	*Lobophora*	Calcite
	*Peyssonellia*	Calcite
	Brown crust	Calcite
*Halimeda*	*Halimeda*	Aragonite
Calcified macroalgae	*Dictyota*	Calcite
	Calcified red macroalgae	Calcite
CaCO_3_	Sediment	Calcite/Aragonite
	Calcium carbonate	Calcite
Fleshy macroalgae	Fleshy red r macroalgae	NC
	Fleshy green algae	NC
	Cyanophyte	NC
Turf	Sponge-turf matrix	NC
	Sediment-turf matrix	NC
	Mixed turf	NC
	Filamentous brown algae	NC
	Filamentous green algae	NC
	Filamentous red algae	NC
NON-CAL	Fleshy inverts	NC
	Colonial tunicate	NC
	Fleshy tubeworm	NC
	Solitary tunicate	NC
	Sponge	NC
	Small tubeworms	NC
	Egg mass	NC
	Biofilm	NC
	Other	NC

### Assessment of biotic parameters in the study sites

Percent benthic cover at each REA site was estimated implementing the Line-Point-Intercept (LPI) methodology at 20 cm intervals along two 25 m line transects set in a single file row (separated by 5 m) at the time of CAU recovery. Live benthic elements, including coral, macroalgae, and other sessile invertebrates were identified to the lowest taxonomic level possible. In addition, at the time of CAU retrieval, benthic communities surrounding each CAU site were photo-documented along the two 25 m transect lines (Table 1). A total of 32 digital images were taken at each site at an elevation of approximately 1 m from the surface of the substrate; these images provided a total sample area of 12 m^2^. Each image was analyzed using Coral Point Count with Excel extensions (v. 4.12) image analysis software (10 stratified random points analyzed per image) [55]. Macroscopic taxa were identified to functional group following an analogous classification scheme as to that implemented for the taxa recruited onto the CAU plates (Table 2). Based on this image analysis, the relative percent cover contribution of each CaCO_3_ polymorph (aragonite, calcite, or high-Mg calcite) of the reef benthos was also calculated by categorizing the calcifying taxa according to their mineralogy [29].

### Assessment of abiotic parameters: water sampling

Discrete water samples were collected by SCUBA divers using a 5 L Niskin bottle directly above the benthos at each REA site during recovery of the CAUs. Thus, water was collected at the depth of the CAU deployment sites. In concert with the water collection, a Seabird 19plus conductivity-temperature-depth (CTD) hydrocast was conducted to characterize the water salinity above the CAU deployment site at the time of discrete water sample collection. Upon completion of the CAU recovery/water sample dive, one 500 ml water subsample from the Niskin bottle was immediately collected and preserved for analysis of total dissolved inorganic carbon, total alkalinity, salinity, dissolved inorganic nutrients, and chlorophyll-*a*. Water samples were stored onboard the NOAA Ship *Hi’ialakai*, following established published techniques [56], and were analyzed at various NOAA and academic institutions within 7 to 60 days following the completion of Pacific RAMP research cruise (Table 3).

**Table 3:** Water chemistry parameters from discrete water samples collected at the study sites during CAU recovery. DIC: dissolved inorganic carbon.

Archipelago	Island/Atoll	Site	Chl-a (μg/L)	PO_4_^3–^ (μM)	Si(OH)^4–^ (μM)	NO_3_^–^ (μM)	NO_2_^–^ (μM)	NO_3_^–^+NO_2_ (μM)		DIC	Total Alkalinity	Salinity
**Pacific**	Baker	**BAK02**	0.099	0.119	1.092	0.286	0.030	0.316	1914.632		2256.760	34.393
**Remote**		**BAK11**	0.142	0.143	1.008	0.289	0.037	0.326	1912.032		2246.860	34.229
**Island**		**BAK14**	0.052	0.178	1.142	0.270	0.036	0.306	1899.453		2237.490	34.198
**Areas**		**BAK16**	0.118	0.176	1.148	0.134	0.031	0.165	1907.366		2243.360	34.215
	Howland	**HOW05**	0.061	0.225	1.152	0.234	0.040	0.273	1923.141		2251.400	34.243
		**HOW11**	0.057	0.261	1.168	0.311	0.044	0.355	1919.946		2255.880	34.330
		**HOW12**	0.047	0.194	1.147	0.175	0.028	0.203	1907.032		2250.920	34.262
		**HOW13**	0.052	0.170	1.088	0.129	0.028	0.157	1904.506		2247.430	34.250
	Jarvis	**JAR01**	0.128	0.381	1.247	3.457	0.176	3.633	2020.984		2329.840	35.477
		**JAR08**	0.085	0.399	1.862	3.895	0.186	4.081	2021.194		2326.440	35.444
		**JAR10**	0.099	0.373	1.306	3.658	0.172	3.830	2018.226		2325.900	35.471
		**JAR11**	0.085	0.441	2.163	4.335	0.167	4.502	2024.863		2324.820	35.441
	Johnston	**JOH09**	0.198	0.835	1.069	0.281	0.056	0.338	1960.300		2257.980	35.186
		**JOH10**	0.080	0.193	0.915	0.164	0.018	0.182	1984.282		2304.380	35.076
		**JOH11**	0.047	0.159	0.895	2.406	0.057	2.462	1987.198		2303.430	35.058
	Kingman	**KIN03**	0.128	0.281	1.304	1.751	0.099	1.850	1949.211		2259.080	34.841
		**KIN04**	0.425	0.226	1.167	0.652	0.071	0.723	1964.640		2285.170	34.806
		**KIN05**	0.345	0.254	1.061	1.265	0.074	1.339	1966.420		2264.910	34.822
		**KIN07**	0.146	0.340	1.381	2.112	0.098	2.210	1969.447		2261.740	34.805
		**KIN10**	0.104	0.316	1.362	2.299	0.090	2.389	1967.024		2251.430	34.816
		**KIN11**	0.146	0.240	1.240	1.008	0.081	1.089	1963.311		2281.420	34.810
		**KIN13**	0.113	0.305	1.329	1.646	0.141	1.788	1971.513		2276.060	34.840
		**KIN16**	0.065	0.248	1.219	1.050	0.101	1.151	1956.153		2276.403	34.815
	Palmyra	**PAL01**	0.132	0.254	1.207	2.157	0.176	2.333	1997.999		2285.630	34.893
		**PAL05**	0.179	0.318	0.967	1.912	0.161	2.073	1972.856		2289.030	34.928
		**PAL11**	0.071	0.323	1.367	3.520	0.122	3.642	1953.555		2249.800	34.793
		**PAL12**	0.189	0.318	1.137	1.976	0.168	2.144	1976.987		2288.100	34.926
		**PAL19**	0.137	0.338	1.065	2.363	0.185	2.548	1980.134		2287.060	34.938
		**PAL21**	0.217	0.313	1.054	1.983	0.164	2.147	1973.778		2289.080	34.924
		**PAL25**	0.061	0.274	1.331	2.559	0.202	2.761	1978.131		2283.410	34.933
		**PAL26**	0.189	0.271	1.524	2.147	0.160	2.308	1982.910		2284.640	34.927
**American**	Ofu-	**OFU01**	0.028	0.174	0.987	0.686	0.026	0.711	1980.514		2326.340	35.548
**Samoa**	Olosega	**OFU02**	0.090	0.193	0.964	0.703	0.021	0.724	1993.481		2342.730	35.671
		**OFU03**	0.104	0.196	0.954	0.323	0.016	0.339	1988.740		2322.960	35.507
		**OFU04**	0.061	0.178	0.908	0.609	0.022	0.631	1983.488		2337.600	35.661
		**OFU06**	0.085	0.227	1.039	0.441	0.022	0.463	2000.860		2347.800	35.722
		**OFU09**	0.038	0.165	0.939	0.201	0.020	0.221	1982.082		2337.270	35.554
		**OLO01**	0.104	0.211	0.835	0.642	0.027	0.669	1980.276		2320.360	35.461
		**OLO04**	0.033	0.162	1.017	0.176	0.012	0.188	1980.092		2338.080	35.637
		**OLO05**	**0.043**	**0.168**	0.969	0.455	0.022	0.477	1987.800		2321.530	35.540
	Rose	**ROS01**	0.024	0.143	1.381	0.734	0.055	0.789	1989.284		2351.650	35.821
		**ROS03**	0.024	0.160	0.982	1.248	0.054	1.302	1997.380		2353.190	35.832
		**ROS04**	0.038	0.213	0.855	0.501	0.023	0.525	1998.827		2352.570	35.824
		**ROS06**	0.033	0.159	0.952	0.660	0.022	0.682	1991.501		2357.540	35.824
		**ROS07**	0.057	0.165	0.930	1.013	0.038	1.051	1993.537		2351.890	35.797
		**ROS08**	0.274	0.173	0.568	0.009	0.007	0.016	1992.341		2345.350	35.813
		**ROS09**	0.387	0.179	0.682	0.030	0.021	0.052	1995.747		2350.240	35.808
		**ROS19**	0.024	0.127	0.960	0.607	0.020	0.626	1990.731		2356.380	35.830
		**ROS23**	0.043	0.235	0.997	0.505	0.022	0.527	1995.147		2350.060	35.830
		**ROS25**	0.043	0.185	0.927	0.830	0.056	0.886	1993.840		2338.330	35.800
	Swains	**SWA01**	0.057	0.171	0.916	0.442	0.025	0.466	1976.990		2324.600	35.377
		**SWA03**	0.047	0.157	0.893	0.419	0.029	0.448	1985.097		2315.480	35.380
		**SWA07**	0.024	0.131	0.883	0.193	0.016	0.209	1966.418		2325.240	35.359
		**SWA08**	0.043	0.110	0.503	0.173	0.013	0.187	1965.466		2321.030	35.359
		**SWA16**	0.031	0.163	0.866	0.464	0.029	0.493	1983.123		2320.943	35.421
	Tau	**TAU02**	0.033	0.178	1.797	0.203	0.012	0.215	2002.217		2345.500	35.587
		**TAU04**	0.047	0.148	1.026	0.157	0.008	0.165	2004.007		2334.260	35.632
		**TAU07**	0.033	0.164	0.900	0.216	0.013	0.229	1977.969		2332.520	35.484
		**TAU08**	0.033	0.174	2.514	0.215	0.010	0.226	1979.494		2337.970	35.559
		**TAU09**	0.090	0.154	1.775	0.158	0.015	0.173	1997.330		2355.710	35.784
		**TAU11**	0.061	0.179	0.981	0.245	0.012	0.257	1984.880		2342.220	35.598
		**TAU12**	0.099	0.265	1.094	0.323	0.020	0.343	2004.229		2352.890	35.868
	Tutuila	**TUT01**	0.250	0.191	1.244	0.463	0.043	0.506	1981.687		2309.060	35.419
		**TUT02**	0.397	0.196	1.149	0.118	0.022	0.140	1983.039		2334.540	35.455
		**TUT05**	0.151	0.157	1.083	0.480	0.035	0.515	1967.571		2314.790	35.371
		**TUT06**	0.076	0.217	1.177	1.202	0.072	1.274	1988.110		2309.950	35.385
		**TUT08**	0.118	0.228	1.399	0.492	0.041	0.533	1982.673		2313.040	35.291
		**TUT09**	0.179	0.203	1.276	0.193	0.033	0.226	1965.812		2322.180	35.477
		**TUT10**	0.231	0.221	1.547	0.693	0.052	0.745	1976.364		2314.330	35.423
		**TUT13**	0.189	0.171	1.412	0.377	0.031	0.408	1967.817		2314.630	35.246
		**TUT14**	0.146	0.171	1.221	0.534	0.042	0.577	1978.316		2312.230	35.346
		**TUT16**	0.090	0.140	0.909	0.501	0.027	0.528	1977.106		2328.470	35.461
		**TUT17**	0.113	0.166	0.710	0.563	0.025	0.588	1968.627		2318.640	35.419
		**TUT19**	0.179	0.179	1.275	0.498	0.030	0.528	1962.147		2310.270	35.267
		**TUT22**	0.156	0.211	1.147	0.341	0.026	0.367	2011.997		2344.960	35.738

### Data analysis

Spatial patterns of mean accretion rates were tested using several independent ANOVA models. A more comprehensive model was not possible because the sample size among the different levels within factors was unbalanced, precluding the analysis of 3-way interactions. Thus, two-way ANOVAs tested for the interaction between island (n = 11) and reef zone (forereef vs. lagoon), island and wave exposure (leeward vs. windward), and wave exposure and island geomorphology (volcanic vs. carbonate) as factors. Data were square root-transformed to fulfill parametric statistical requirements. The tests of island and wave exposure, and wave exposure and island geomorphology, were run on forereef data only. Additional non-parametric Kruskal-Wallis ANOVAs were implemented to test for differences in percent cover of calcifying taxa between the CAU plates and the benthos, and for spatial differences in percent cover of CCA and macroalgae+turf algae of the CAU plates; pair-wise comparisons (Dunn’s test) were performed to establish differences among islands. Due to the constrains placed by the assumptions of parametric statistics, Spearman Rank Order Correlation tests were implemented to explore the association between: 1) the percent cover of CCA on the CAU plates vs. the benthos; 2) the site-specific mean accretion rates and the percent cover of CCA on the CAU plates; and 3) island mean accretion rates and water chemistry parameters. All ANOVA and correlation analyses above mentioned were performed using SYSTAT 12 version 12.02.00 [57].

To further explore the combined effects of the biotic and abiotic parameters a Regression with Empirical Variable Selection Procedure (hereafter REVS) was employed to identify models that best predicted the spatial variability in carbonate accretion rates across reefs in the study area. The REVS procedure evaluates all possible regression models (i.e., combination of predictor variables) and displays the best-fitting models that contain one predictor, two predictors, and so on [58]. Because differences in the benthic communities between forereef and lagoon habitats can have an important effects on calcium carbonate accretion mechanisms and rates; lagoon sites (n = 11) and forereef sites (n = 67) were analyzed separately. Two sets of predictor variables were evaluated to investigate relationships with carbonate accretion rates: (1) biotic; i.e, the percent cover of benthic organisms in the benthic transect survey dataset and (2) abiotic; i.e, water quality parameters The relationship between the remaining predictor variables for each set, and carbonate accretion rates were then analyzed using Generalized Additive Models (GAM). All carbonate accretion rate analyses were performed in R (R Development Core Team, 2014) using the packages "agricolae", "car", "doBy", "leaps", "MASS", "mgcv", "pgirmess", "plyr", "reshape", "stringr" as well as the non-packaged R function "REVS" [58].

Finally, to determine the similarity between the overall percent cover of the organisms on CAU plates and the overall percent cover of the organisms on the benthos, a RELATE test was conducted using PRIMER v.6. This test performs a series of non-parametric correlations between all elements within each of the two data sets. If the among-sample relationships agree in exactly the same way in both data sets, then the overall rank correlation rho-value (ρ) = 1, perfect match; values closer to zero indicate little to no overall similarity between the two data sets [59, 60]. Prior to analysis, raw percent cover data were consolidated into functional groups [i.e., biofilm, scleractinian coral, calcified invertebrates (excluding scleractinian coral), fleshy invertebrates, CCA, fleshy encrusting macroalgae, calcified encrusting macroalgae (excluding CCA), fleshy upright macroalgae, calcified upright macroalgae (excluding *Halimeda*), *Halimeda*, turf algae, empty CAU tile, unidentifiable CaCO_3_, loose sediment; (Table 2)]; analyses were limited to sites having both LPI and CAU cover data sets (see Table 1). Data from CAUs was averaged by site (n = 4 or 5) and utilized structural composition data from the top plate only. Both the LPI and CAU data were square root-transformed to reduce the influence of abundant functional groups and increase the influence of less common groups, and resemblance matrices were created using Bray-Curtis similarity. The RELATE test was used on the LPI and CAU data matrices based on Spearman rank correlation method with 9,999 permutations. A result rho-value (ρ) close to 1 would indicate high similarity in patterns of ranked order abundance between the LPI and CAU matrices, while a value close to zero would indicate little similarity.

## Results

### Accretion rates

**Fig 3:**
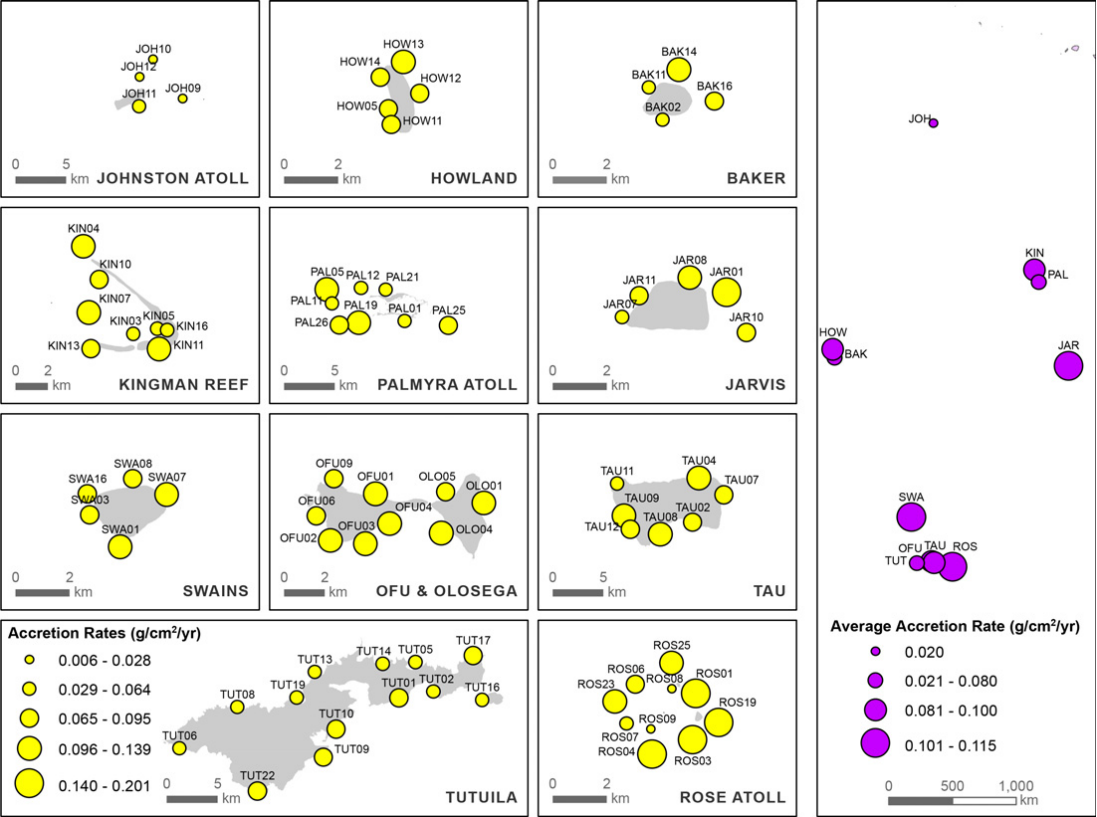
Spatial distribution and mean carbonate accretion rates derived from CAU deployments by study site (left panel) and island-wide (right panel).

**Fig 4:**
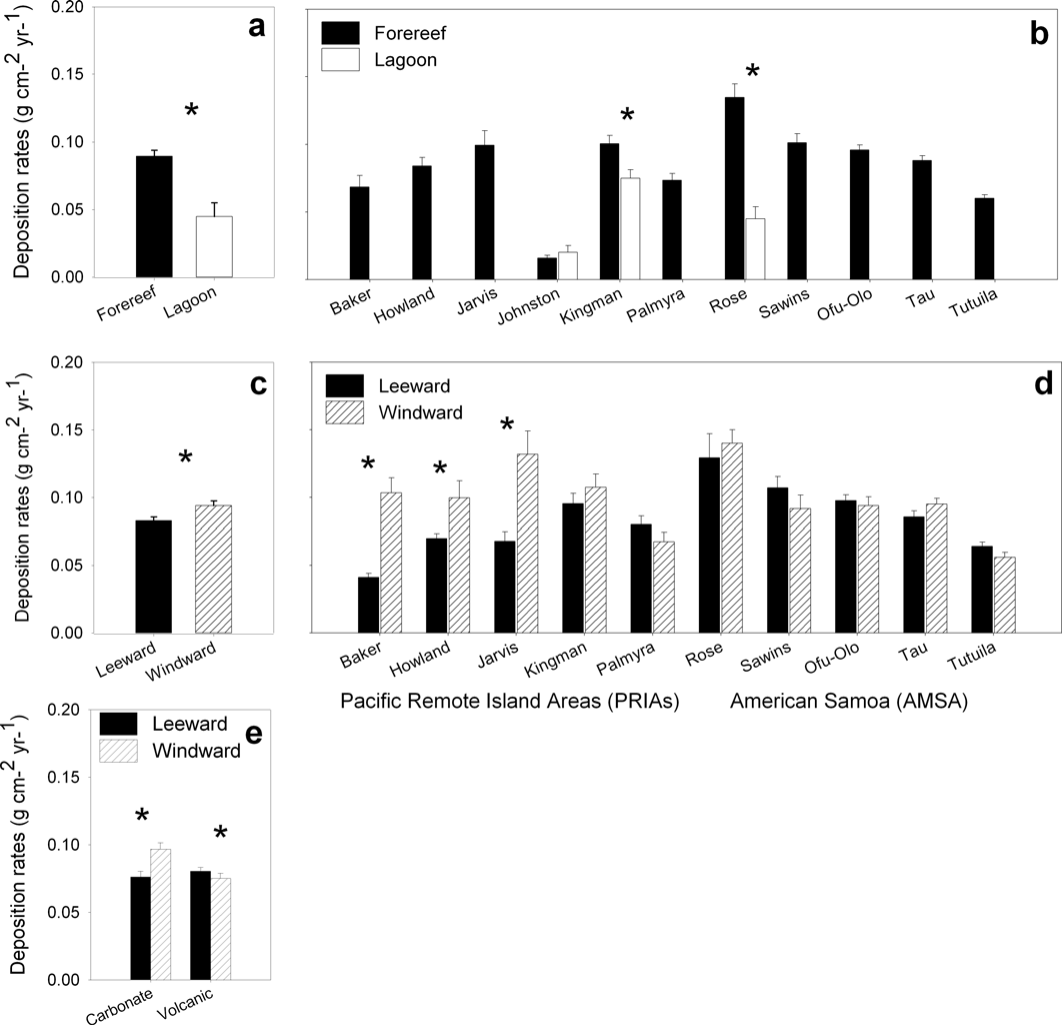
Graphic representation of the independent ANOVA model results illustrating the spatial variation patterns in net CaCO_3_ accretion rates among island and reef zones (a, b); islands and levels of wave exposure (c, d), and island geomorphology (volcanic vs. carbonate) and wave exposure (e). Islands graphed in order of latitude; asterisks indicate significant differences among bar pairs.

Of the 390 CAUs deployed, 365 were recovered (94%); missing CAUs occurred haphazardly across a variety of sites including forereef, lagoon, sheltered, and exposed sites. Rose Atoll and Ta`u had the highest percentage of missing CAUs, with 10 and 15% of units missing, respectively. Net accretion rates varied across a wide range of spatial and environmental constructs including reef zone (forereef vs. lagoon), latitude, island, exposure (leeward vs. windward), population (urban settlements vs. none), geomorphology (carbonate vs. volcanic), and sites (Fig 3). Individual CAU accretion rates varied by orders of magnitude; they ranged from 0.004 g CaCO_3_ cm^-2^ yr^-1^ at JOH-10, a lagoon site at Johnston Atoll, to 0.251 g CaCO_3_ cm^-2^ yr^-1^ at JAR-01 on the forereef at Jarvis Island. Of the 78 sites examined, average accretion rates differed between islands (n = 11) and reef zones (forereef vs. lagoon). There was no interaction, but each factor had a significant main effect, with rates being significantly greater at forereef sites compared to lagoon sites (Fig 4A and 4B) (two-way ANOVA; F_ISLAND_ = 16.19, *df* = 10, p<0.001; F_REEF ZONE_ = 33.13, *df* = 1, p<0.01) (Table 4). Differences among islands exhibited a spatial pattern according to latitude; the equatorial reef systems at Howland, Baker, Jarvis, Palmyra, and Kingman Reef exhibited comparable accretion rates, with no statistical differences among them. Contrastingly, significantly different levels of variability were evident among the higher-latitude reef systems, with Tutuila exhibiting the lowest rates and Rose Atoll the highest (p<0.001, Tukey pairwise multiple comparison); no differences were evident between Swains, Ta`u, and Ofu-Olosega (p>0.05, Tukey pairwise multiple comparison). CaCO_3_ accretion rates at these higher-latitude islands (Ofu-Olosega, Ta`u, and Swains) did not differ from the equatorial reef systems above mentioned (p>0.05, Tukey pairwise multiple comparison). In addition, urbanization and human inhabitation did not have a clear effect on the inter-island patterns of CaCO_3_ accretion. Although Johnston Atoll, Palmyra Atoll, and Tutuila exhibited the lowest island/atoll-wide accretion rates and coincidentally have undergone the greatest levels of human disturbance (extensive dredging, morphological changes, deforestation, land-based sources of pollution, and nuclear and biological weapons testing), the pattern of inhabitation/high disturbance regime and low accretion rates was not consistent for other inhabited islands such as Swains, Ta`u and Ofu-Olosega or historically human impacted reef systems such as those at Howland, Baker, and Jarvis. This is likely due to the low levels of human inhabitation at Swains, Ta`u and Ofu-Olosega (population = 17, 790, and 358, respectively) (U.S. Census Bureau 2010[61]. Considering forereef sites only, accretion rates differed significantly among islands and levels of wave exposure (leeward vs. windward), but no interaction effects among factors were detected, with rates being significantly greater at windward sites compared to leeward sites (Fig 4C and 4D) (two-way ANOVA; F_ISLAND_ = 14.18, *df* = 10, p<0.001; F_EXPOSURE_ = 4.91, *df* = 1, p = 0.027) (Table 4). For the main effect of islands and exposure, this difference was only statistically significant at equatorial and topographic-upwelling islands of Baker, and Jarvis. Finally, the third two-way ANOVA using island geomorphology (volcanic vs. carbonate) and exposure (leeward vs. windward) revealed a significant interaction between these factors (two-way ANOVA; F_EXPOSURE × GEOMORPH_ = 9.66, *df* = 1,1; P = 0.002) (Table 4). At carbonate islands, accretion was higher at windward compared to leeward sites, but rates were equivalent at both exposures on volcanic islands (Fig 4E).

**Table 4:** Summary results (F, χ^2^, R, and P values) of all independent ANOVA and correlation statistical tests run to analyze accretion, percent cover, and water chemistry data.

Test	F	χ^2^	R	P
**Two-way ANOVA: Mean net accretion rates**				
Island	16.19			< 0.001
Reef zone	33.13			< 0.001
Island x Reef zone	No interaction effects			
Island	14.18			< 0.001
Exposure	4.91			0.027
Island x Exposure	No interaction effects			
Exposure	0.49			0.48
Geomorphology	3.02			0.08
Exposure x Geomorphology	9.66			0.002
**Kruskal-Wallis ANOVA**				
Mean % cover of calcifiers (CAU vs. benthos)		13.31		<0.001
Mean % CCA cover on CAUs/Islands		97.97		< 0.001
Mean % Turf + macroalgal cover on CAUs/Islands		44.42		< 0.001
**Spearman Rank Order Correlations**				
Mean % CCA cover on CAUs vs. accretion rates			0.64	< 0.001
Mean % CCA cover on benthos vs. accretion rates			0.42	< 0.001
Mean % CCA cover on CAUs vs. benthos			0.44	<0.001
Island mean accretion rates vs. TA			0.30	<0.01
Island mean accretion rates vs. Chl-a			‒0.47	<0.001
Island mean accretion rates vs. PO_4_^3−^			‒0.13	>0.05
Island mean accretion rates vs. Si(OH)^4−^			0.03	>0.05
Island mean accretion rates vs. NO_3_^−^			0.10	>0.05
Island mean accretion rates vs. NO_2_^−^			0.22	>0.05
Island mean accretion rates vs. NO_3_^−^+NO_2_^−^			0.10	>0.05
Island mean accretion rates vs. DIC			0.21	>0.05
Island mean accretion rates vs. Salinity			0.29	0.01

### Community composition and percent cover

**Fig 5:**
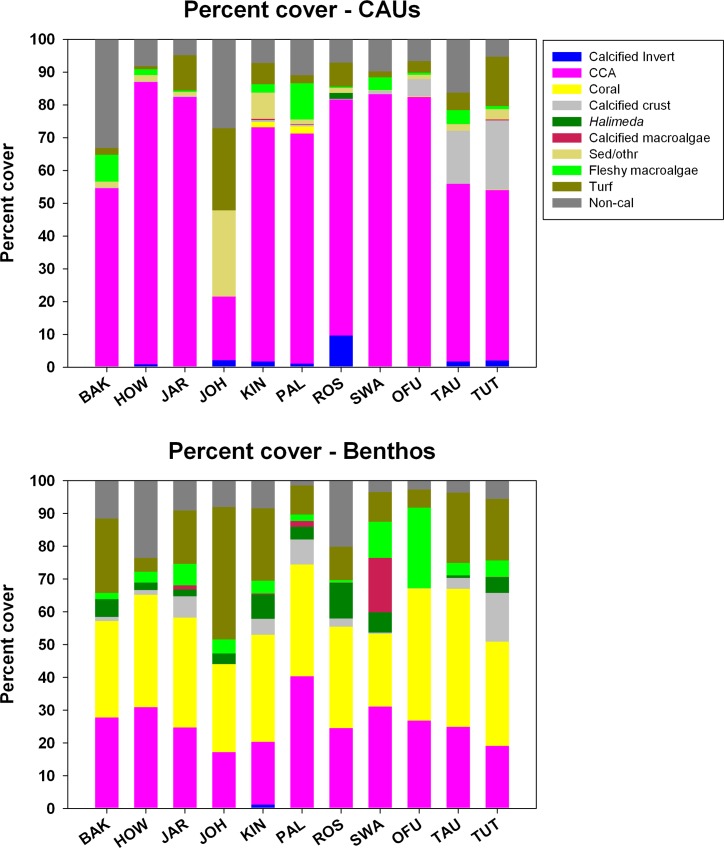
Percent cover of the upper CAU plate and the surrounding site benthos derived from image analysis and LPI surveys (see methods for details) and classified by functional groups: Calcified invertebrate; CCA: crustose coralline algae; coral; calcified algal crusts; *Halimeda*; calcified macroalgae; sediments/other; fleshy macroalgae; turf; and non-calcified material. BAK: Baker Island; HOW: Howland Island, JAR: Jarvis Island; JOH: Johnston Atoll; KIN: Kingman Reef; PAL: Palmyra Atoll; ROS: Rose Atoll; SWA: Swains Island; OFU: Ofu and Olosega Islands; TAU: Ta`u Island; and TUT: Tutuila Island.

Mean island-wide percent cover of the major calcifying organisms on the reef benthos and those that recruited to the top surface of upper CAU plates are contrasted in Fig 5. Overall, the percent cover of calcifying to non-calcifying taxa differed between the CAU plates and the benthos (78.4% ± 2.2 and 68.7% ± 1.8, respectively; Kruskal-Wallis ANOVA, χ^2^ = 13.31, *df* = 1, p<0.001) (Table 4), as well as the proportion of cover represented by each of the different calcifying functional groups. For example, for all sites combined, CAUs were dominated by CCA (66%), with a lesser contribution by CaCO_3_ sediment (4.4%), and calcified algal crusts (4.1%). This contrasts with the benthic communities at the deployment sites, where the major calcifying taxa included scleractinian corals (32%), CCA (26%), and calcified algae (6% predominantly *Halimeda* and Peyssonneliales). For all reef systems with the exception of Johnston Atoll, CCA represented more than 50% of cover on the CAU plates and differences in CCA cover among islands were statistically significant (Kruskal-Wallis ANOVA, χ^2^ = 97.97, *df* = 10, p<0.001) (Table 4). Interestingly, the community composition on the CAU plates for Johnston and Tutuila exhibited a greater proportion of fleshy macroalgae and turf algae combined (Mean ± SE: 25.1% ± 6.1; 16.1% ± 2.9, respectively), compared to the other islands and atolls (7.1% ± 0.9), and those differences were statistically significant (Kruskal-Wallis ANOVA, χ^2^ = 44.42, *df* = 10, p<0.001; Dunn’s Test pairwise multiple comparisons) (Table 4).

### Biotic and abiotic correlates

Percent cover of CCA on CAUs was significantly correlated with net accretion rates (r = 0.64, p<0.001; Spearman Rank Order Correlation), as was CCA cover of the benthos (r = 0.42, p<0.001; Spearman Rank Order Correlation) (Table 4). Despite a significant association between percent cover of CCA on the CAU plates and the benthos (r = 0.44, Spearman rank order correlation) the RELATE analysis indicated that overall benthic communities found on CAU plates did not closely resemble what was found on the surrounding substrate, this was clearly evient from the low rho-value (ρ = 0.243). We also found a positive statistical association between mean accretion of CCA and *in situ* total alkalinity and salinity (r = 0.30, p<0.001 and r = 0.29, p = 0.01, respectively; Spearman Rank Order Correlation), and a negative statistical association with chlorophyll-*a* concentration (r = −0.47, p<0.001; Spearman Rank Order Correlation); mean Island accretion rates exhibited non-significant correlations all the other water chemistry parameters (Table 4).

The optimal abiotic REVS model corroborated the results from the independent correlation tests above. As such, the spatial variability in the carbonate accretion rates on forereefs was best explained by two environmental predictor variables: total alkalinity and chlorophyll-a (r = 0.33, p = 0.0079 and r = −0.4, p = 0.001, respectively; REVS). In the subsequent GAM analysis, only total alkalinity was retained as the explanatory variable. For the lagoon sites, the optimal abiotic REVS model contained two environmental predictor variables that were positively associated with the carbonate accretion rates: silicon hydroxide (dissolved silica; r = 0.77, p = 0.0095) and dissolved inorganic carbon (r = 0.82, p = 0.0041). In the subsequent GAM analysis, only dissolved silica was retained as a statistically significant predictor variable. The biotic variables to best predict the spatial variation in carbonate accretion rates on forereefs included CCA cover and coral cover (r = 0.54, p<0.001; r = −0.03, p = 0.824, respectively; REVS) however, due to the low level of association only CCA was retained as statistically significant predictor in the GAM analysis. Finally, for the lagoon sites, the optimal biotic REVS model identified four explanatory variables; two were positively correlated with carbonate accretion rates [*Halimeda* (r = 0.69, p = 0.0197) and non-coralline encrusting macroalgae (r = 0.15, p = 0.6583)] and two were negatively correlated turf algae (r = −0.47, p = 0.1456) and fleshy upright macroalgae (r = −0.48, p = 0.1329)]. Of these, *Halimeda* was the only statistically significant variable retained in the GAM analysis.

### Carbonate mineralogy

**Fig 6:**
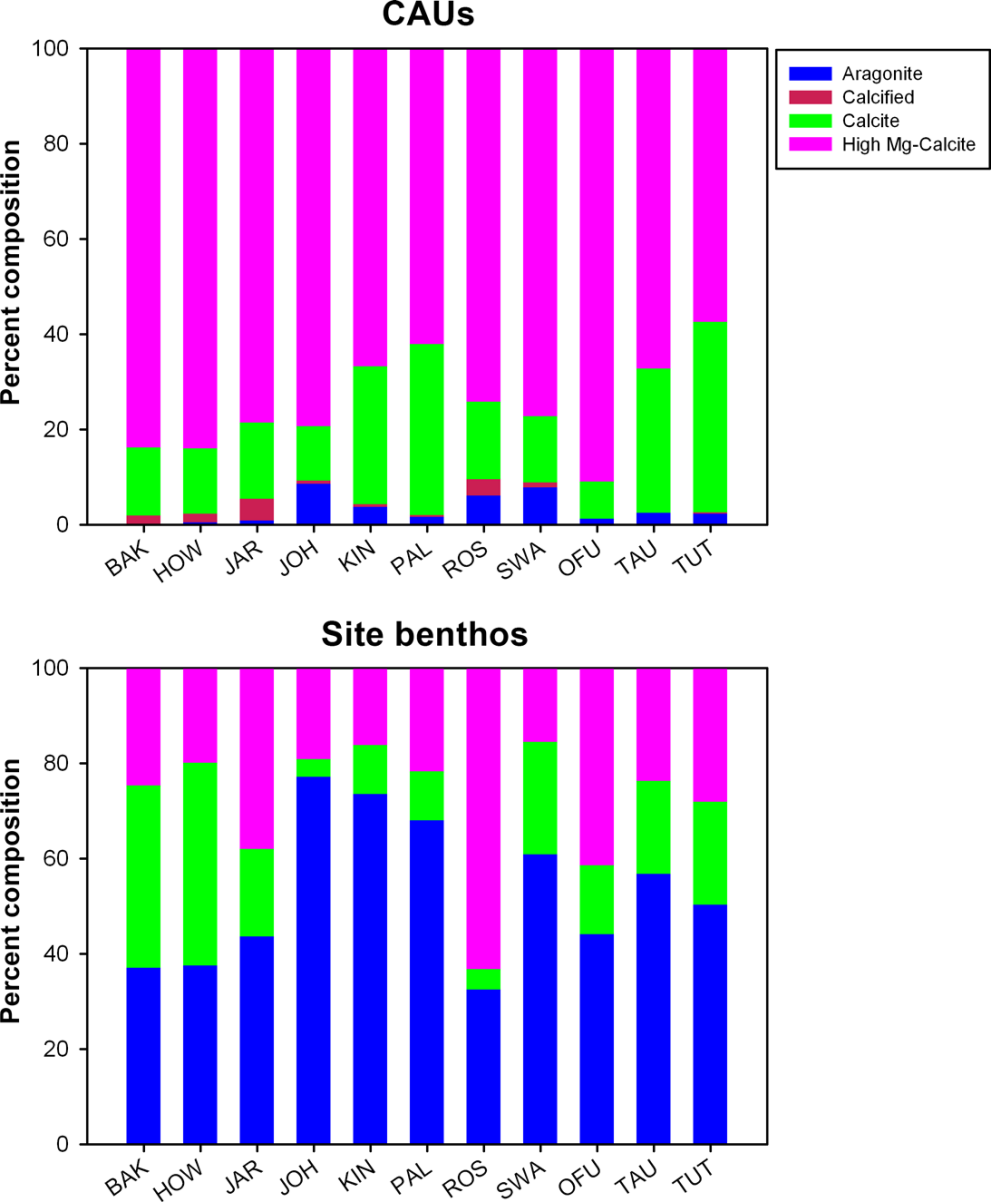
Percent composition of the various CaCO_3_ polymorphs computed based on the in-situ percent cover of the different functional groups recruited to the CAU plates and on the mature benthos (see methods for details). Calcified: unidentified calcified material. BAK: Baker Island; HOW: Howland Island, JAR: Jarvis Island; JOH: Johnston Atoll; KIN: Kingman Reef; PAL: Palmyra Atoll; ROS: Rose Atoll; SWA: Swains Island; OFU: Ofu and Olosega Islands; TAU: Ta`u Island; TUT: Tutuila Island.

When net site-specific accretion rates were combined with the percent cover of the different functional groups of known mineralogy recruited to the CAUs and on the benthos, high Mg-calcite was found to be the dominant carbonate polymorph of the reef early successional stages. For oceanic reef systems such as Howland, Baker, Jarvis, Johnston, Swains, Rose, and Palmyra, the net accretion of organisms depositing high Mg-calcite represented over 70% on the CAU plates, compared to ~30% on the benthos (Fig 6). These differences are to be expected, given that CCA was the major calcifying functional group recruiting to the CAU plates, in contrast to the reef benthos where organisms depositing aragonite (scleractinian corals, milleporids, and *Halimeda*) out-weighed those depositing high Mg-calcite.

## Discussion

This study presents a comprehensive, quantitative assessment of the rates of net CaCO_3_ accretion *in situ* across a diverse range of reef systems in the central and south Pacific and demonstrates that: 1) net carbonate accretion rates of early reef successional stages varied considerably across a wide range of spatial and environmental constructs, including island, site, reef zone, latitude, exposure (leeward vs. windward), population (urban settlements vs. none), and geomorphology (volcanic vs. carbonate); 2) CCA benthic percent cover of the surrounding benthos, total alkalinity, and chlorophyll-a concentrations were significant predictor variables for net carbonate accretion rates on forereef habitats, and dissolved silica and percent cover of *Halimeda* were the significant predictor variable for lagoon habitats, respectively; and 3) the composition and relative abundance of the key early successional taxa recruited on to CAUs differed from that of the surrounding, mature benthos, with the former being overwhelmingly dominated by crustose coralline algae (CCA; >70% cover). The results of this study also provide insight into CaCO_3_ accretion rates on standardized surfaces across an anthropogenic gradient, from relatively undisturbed, quasi-pristine coral reefs to human impacted (see [62]).

The large range of accretion rates within and among islands are likely the result of the complex and spatially variable nature of the physical and biological processes driving the structure and function of reef communities. Overall, accretion rates were higher on forereef sites than in lagoon habitats because of the higher amount of CCA present on CAUs from these reef zones. Although the lagoon environments at Johnston, Rose, and Kingman Reef are very different from each other, the observed forereef vs. lagoon differences are likely driven by key coral reef community structural determinants, including depth, light availability, wave exposure, as well as, the disparate levels of water circulation and flushing, turbidity and sedimentation, and productivity that characterize each reef zone [63, 64, 65]. The effect of exposure (leeward vs. windward), was only manifest for the three equatorial islands in the PRIA (Baker, Howland, and Jarvis). This difference is at least partially due to the intense topographic upwelling of the Equatorial Undercurrent on the west side of all three equatorial islands; upwelling brings more nutrients, reduced light penetration, and sedimentation of organic particles [50]. CCA are photosynthetic organisms that require adequate light levels to calcify and grow; in addition high phosphate concentrations have been demonstrated to be detrimental to CCA development [66]. Moreover, the leeward environs on the three equatorial islands above-mentioned are characterized by steep-sloping forereefs compared to the windward facing habitats which are typified by broad shallow, forereef terraces [65, 67]. Shading on the steep leeward reef slope could also contribute to lower levels of carbonate accretion for these areas, whereas reef communities on the broad shallow forereef terraces of windward shores received full sun exposure.

With the exception of Palmyra Atoll, the equatorial reef systems at Howland, Baker, Jarvis, and Kingman, exhibited comparable net carbonate accretion rates. In contrast, carbonate accretion rates in American Samoa and Johnston Atoll exhibited notably high levels of variability. Johnston and Tutuila at 16°N and 14°S, respectively, had the lowest average accretion rates (0.019 g CaCO_3_ cm^-2^ yr^-1^ and 0.060 g CaCO_3_ cm^-2^ yr^-1^, respectively) and Rose Atoll the highest (14°S, 0.116 g CaCO_3_ cm^-2^ yr^-1^); accretion rates for the islands of Swains, Ta`u, and Ofu-Olosega were comparable to each other (0.09–0.100 g CaCO_3_ cm^-2^ yr^-1^). Interestingly, the three reef systems exhibiting the lowest average accretion rates (i.e., Johnston Atoll, Palmyra Atoll, and Tutuila) have also, historically, experienced the highest levels of human impact. For example, Johnston and Palmyra atolls were extensively dredged and substantially modified to accommodate the operation of military naval bases and air strips during the WWII U.S. Pacific campaign. Some of these alterations resulted in widespread, chronic changes to water clarity and circulation, in addition to more recent human disturbances including localized iron-leaching from ship groundings and PCB contamination [67].

For Tutuila, increasing anthropogenic impacts resulting from significant human inhabitation and subsequent urban development have degraded water quality in many reef habitats around the island, particularly due to runoff carrying considerable amounts of sediments and nutrients [68]. Higher nutrient levels facilitate the proliferation of fast-growing macroalgae and turf algae, which in turn can easily out-compete reef calcifiers for space and resources. Concomitantly, increased runoff generally results in reductions in water clarity, which in turn can negatively affect the net carbonate accretion rates, given that the main reef calcifiers are photosynthetic and require clean, well-lit waters [69,70]. Overfishing of reef herbivores, particularly parrotfish and surgeonfish, is an additional result of increasing population pressure at Tutuila [68]. With the loss of grazers, epiphytic filamentous and turf algae can quickly overgrow reef calcifiers and these effects are often exacerbated when increased nutrients are implicated [71]. As such, the combined effects of chronic human disturbances together with decreased pH from ocean acidification will likely affect reef community structure and therefore carbonate accretion on coral reefs worldwide [72].

The spatial variability in CAU net accretion rates at forereef sites was related to total alkalinity (TA). TA, defined as the stoichiometric sum of the bases in solution, is a measure of the capacity of water to resist changes in pH. In tropical reef ecosystems TA is predominantly governed by the concentration of the carbonate ion (CO_3_^2−^) in seawater; benthic and water-column processes, including biological calcification and photosynthesis can drive site-level changes in carbonate ion concentrations [47, 73, 74]. As such, a positive, statistical association between TA and net accretion rates is expected because higher pH and TA conditions shift the carbonate system balance to thermodynamically favor CaCO_3_ precipitation. In addition, Chl-a concentration was the optimal biotic predictor variable of net accretion rates at forereef sites. As previously mentioned, high nutrient concentrations, in particularly phosphate, have a detrimental effect of CCA calcification and growth [66]. Because Chl-a concentration is a proxy for ocean photosynthetic productivity, which in turn is affected by nutrient availability [50], a negative statistical association with accretion rates would be expected. The significant association between accretion rates and percent CCA benthic cover is also expected given that CCA was the overall greatest contributor to CaCO_3_ accretion rates of early reef successional stages.

For the lagoon sites, two environmental variables were positively correlated with the carbonate accretion rates: dissolved silica and dissolved inorganic carbon (DIC), of which only dissolved silica was retained in the GAM analysis as a significant predictor variable. This finding is consistent with the selection of percent *Halimeda* benthic cover as the sole biotic predictor variable that correlated significantly and positively with carbonate accretion rates. Although *Halimeda* is one of the major carbonate producers in tropical reef systems [32], it was rare or completely absent from all the lagoon sites at Rose and Johnston atolls, and only moderately high at two outer lagoon sites at Kingman Reef. The statistical associations between the predictor variables and the carbonate accretion reflect the pattern of relatively high accretion rates at the two lagoon sites at Kingman reef (KIN-07 and KIN-10) and substantially low accretion rates at the remaining lagoon sites (Fig 3). While the intrinsic drivers of these associations remain unclear, we suggest that the low sample size (n = 10) in concert with the marked structural and ecological differences between the three lagoon systems (e.g., open lagoons at Kingman and Johnston vs. closed lagoon at Rose Atoll; relatively pristine conditions at Rose Atoll and Kingman Reef vs. extensive anthropogenic impacts at Johnston Atoll) may be in part implicated in the spatial patterns reported herein; further study is recommended.

The average percent cover of the main benthic components at the study sites was approximately: 33% for scleractinian corals, 26% for CCA, and 16% for turf algae; on the CAU plates these taxa represented 0.4%, 70%, and 7%, respectively. It is the disparate proportions in percent cover of the key early successional taxa on CAU plates and the mature benthos the main reason why the RELATE analysis showed little similarity between CAU plates and the surrounding benthos. This can be explained in part due to the short deployment period (2 years) of the experimental units. Nonetheless, despite those differences, the spatial variability in carbonate accretion rates reported in this study could be predicted by the combination of biotic and abiotic parameters, demonstrating the utility of CAUs as a monitoring tool for the effects of ocean acidification (OA).

In addition, high Mg-calcite was found to be the dominant carbonate polymorph deposited on the CAU plates. This is expected, given that CCA was the major calcifying functional group recruiting to the CAU plates, in contrast to the reef benthos where organisms depositing aragonite (scleractinian corals, milleporids, and *Halimeda*) out-weighed those depositing high Mg-calcite. Many coralline algal species precipitate high Mg-calcite [75], with the highest molar mass of MgCO_3_ ratio at low latitudes and warm temperatures [11]. High Mg-calcite is the most soluble form of biogenic CaCO_3_, making coralline algae amongst the most susceptible coral reef taxa to ocean acidification [21, 45].

A great deal of emphasis has been devoted to understanding and characterizing the effects of OA on tropical coral reef ecosystems [76]. Nonetheless, despite their pivotal role as major source of reef limestone, reef habitat creation, and their association with the recruitment process of key reef elements including scleractinian corals, insufficient attention has been paid to the potential implications of elevated ocean pCO_2_ to crustose coralline algae [36, 77, 78]. With CCA representing such an important proportion of calcifying biota both on the early reef successional community, as well as the mature reef benthic community, it is clear that the effects of OA can profoundly affect coral reef function at multiple ecological levels: from the recruitment of CCA and the organisms dependent on them for settlement [37], to the production, stabilization, and cementation of the reef framework and carbonate sediments [79, 80].

Our study provides insight into variation in carbonate accretion rates, primarily by CCA, at dozens of sites across the central and south Pacific, and offers a unique perspective to contextualize our comprehension of the effects of OA at different scenarios of future ocean chemistry. As such, three main inferences can be gleaned from our observations: (1) the spatially variable nature of the accretion rates reported herein suggest that reef community responses will likely vary widely between reef systems, but between sites within islands as well; (2) because CCA precipitate a highly soluble polymorph of CaCO_3_, changes in ocean water acidity will likely result in lower CCA accretion rates; and (3) under acidified conditions CCA may lose their competitive advantage as the dominant calcifying taxa of the early reef successional community, which in turn may have adverse implications for the settlement and development of other important reef calcifying taxa. Therefore, under the projected changes in marine seawater carbonate chemistry, the ability of marine biomineralizers to cope with such changes and continue offering the ecosystem services they currently provide will likely be determined by both the magnitude and rate of seawater pH decrease. As such, the combined effects of chronic human disturbances together with decreased pH from ocean acidification will likely affect reef community structure and therefore carbonate accretion on coral reefs worldwide
